# How organizational trust impacts organizational citizenship behavior: Organizational identification and employee loyalty as mediators

**DOI:** 10.3389/fpsyg.2022.996962

**Published:** 2022-11-15

**Authors:** Yuping Dai, Yuk Ming Tang, Weinian Chen, Jie Hou

**Affiliations:** ^1^Faculty of Business, City University of Macau, Macau, Macao SAR, China; ^2^Department of Industrial and Systems Engineering, the Hong Kong Polytechnic University, Kowloon, Hong Kong SAR, China; ^3^School of Foreign Languages, Huaibei Normal University, Huaibei, Anhui, China

**Keywords:** organizational trust, organizational identification, employee loyalty, organizational citizenship behavior, Chinese enterprises

## Abstract

Research on the influence of organizational trust (OT) on organizational citizenship behavior (OCB) of employees has been conducted for years, but the influence of internal mechanism for OT on OCB is not well studied. Based on social exchange theory and organizational identity theory, this paper explored the OT on the OCB and revealed the chain-mediating role of organizational identification (OI) and employee loyalty (EL) from both employees’ cognitive and psychological perspectives. The research employed a two-wave and multi-source strategy to conduct hypothesis validation with 305 validated questionnaires of Chinese enterprises. The results of the empirical analysis show that: (1) OT has a significant effect on OCB; (2) OI plays a mediating role between OT and OCB; (3) EL plays a mediating role between OT and OCB; and (4) OI and EL have a significant chain-mediating role between OT and OCB. The results of this study have deepened the understanding of organizational trust and have important practical implications for improving organizational citizenship behavior, organizational identification, and employee loyalty.

## Introduction

Since the outbreak of COVID-19, the external market environment has changed dramatically, which has brought much pressure to the survival and development of enterprises that intend to maximize their operational efficiency (Kim, 2019). In this situation, the enterprise puts forward higher expectations for the role of employees, and they need to actively engage in tasks beyond their assigned work, such as organizational citizenship behavior (OCB; Ali and Miralam, 2019; Sultana and Johari, 2022). OCB refers to the voluntary commitment of individuals to behaviors beyond their job duties and is often not incorporated into the organizational formal rewards, but it can be effective in promoting organizational operation and plays a non-negligible role in corporate development (Organ and Ryan, 1995; Singh and Srivastava, 2016; Adil et al., 2021). As extra-role behaviors, OCB could function to help new employees to adapt themselves to their own positions as quickly as possible (Adil et al., 2021), aiding the team to solve complicated problems (Marinova et al., 2019; Choong and Ng, 2022). Employee’s OCB offers various positive impacts. For employees, it can improve their sense of self-efficacy (Choong and Ng, 2022), personal reputation, and job satisfaction. For organizations, it can enhance team cohesion (Podsakoff et al., 1990), promote innovation (Marinova et al., 2019), and so on.

How to motivate OCB of employees has been widely concerned by researchers and managers. Studies have found that organizational, leadership, work, and personal factors could influence OCB (Kim and Park, 2019; Marinova et al., 2019). As an important situational element that motivates a firm to function well, organizational trust (OT) is an influential determinant of employees’ work attitudes and behaviors (Altuntas and Baykal, 2010; Saleem et al., 2020). OT can not only provide psychological safety to organizational members, but also encourage employees to express their ideas freely and promote job performance (Verburg et al., 2018). Previous studies mainly explored the relationship between organizational trust and organizational citizenship behavior from a single theoretical perspective, such as social exchange theory (Singh and Srivastava, 2016), affective event theory (Lu, 2014), and social cognitive theory (Choong and Ng, 2022). The findings found that the relationship between OT and employees’ OCB is complex and uncertain. Some studies point out that OT stimulates OCB among employees (Singh and Srivastava, 2016; Kim and Park, 2019; Yildiz, 2019), and there are also results showing that OT negatively affects OCB (Ali and Miralam, 2019). Moreover, Lu (2014) concluded that emotional trust positively promotes OCB, while a cognitive trust does not affect OCB. Therefore, exactly how OT impacts employees’ OCB through mediating mechanisms is still debatable and requires further research discussion.

The process from OT to OCB of employees is a complex conduction mechanism. OT is essentially an organizational climate factor (Li et al., 2021), while OCB of employees is an altruistic behavior generated by an individual’s internal motivation (Organ and Ryan, 1995; Altuntas and Baykal, 2010). From external situational factors to the occurrence of individual behavior, the individual’s cognitive and psychological states are required to play a role of the bridge. However, previous studies neglected the attribution and identification of individuals when discussing the relationship between trust and OCB. Social identity theory provides a theoretical framework to explain the linkage of OT to employees’ OCB (Van Dick et al., 2006). On the one hand, the trust serves as a nexus of close connections between organizational members and is fundamental in facilitating internal organizational relationships (Altuntas and Baykal, 2010; Saleem et al., 2020). An individual’s judgment of the organizational context constitutes a perception of and generates an emotional identity with the organization that is, in effect, the individual’s trust in the organization (Barattucci et al., 2021). On the other hand, OT is an important prerequisite for OCB. Ashforth and Mael (1989) found that employees with high OT perceive the success and failure of the company as their own responsibility and share weal and woe with the organization. Marstand et al. (2021) argued that employees who develop a strong sense of identification with the organization are more likely to display positive, upbeat attitudes to work and spontaneously do something that benefits the organization. Therefore, we suggest that organizational identification (OI) may play a mediating role in the effects of OT on OCB.

Employee loyalty (EL) is a significant attribute to gain competitive advantage in any organization (Dutta and Dhir, 2021). Numerous studies have confirmed that tangible and material factors promote EL, such as promotion, salary, benefits, working conditions, and so on (e.g., Nadeak and Naibaho, 2020; Phuong and Vinh, 2020). In addition to economic factors, the academic and practical communities have called for an emphasis on the importance of non-material factors on EL, such as organizational commitment, employee satisfaction, and the support from leaders (e.g., Phuong and Vinh, 2020; Li et al., 2021; Veloso et al., 2021). According to social exchange theory, human interactions in organizations are essentially a series of exchanges based on the principle of “reciprocity.” When employees perceive a wealth of instrumental and emotional support, they are more willing to devote ingenuity to the development of the organization, build loyalty (Dutta and Dhir, 2021), and then be willing to make more contributions to the organization. Therefore, we believe that when employees perceive the trust from the organization. in turn, they will reward the organization with a more loyal attitude and show more OCB in their work.

By integrating social exchange theory (Cropanzano et al., 2017) and organizational identity theory (Ashforth and Mael, 1989), we provide a better understanding of how OT impacts employees’ OCB by testing the mediating roles of OI and EL in the process of OT impacting OCB. Specifically, our research questions include the following aspects. (1) Is there a connection between the OT, OI, EL, and OCB of employees? (2) Is the relationship between OT and OCB affected by the mediating effects of OI and EL?

This study mainly contributes to the following aspects. (1) The research effectively combined social exchange theory and organizational identity theory to construct a model of the relationship between OT, OI, and EL and the OCB of the employees and empirically examined the current knowledge about the impact of OT on organizational citizenship. (2) The paper shed light on the relationships among OT, OI, EL, and OCB through empirical research, which can provide guidance and reference for firms to develop corresponding measures to motivate employees’ OCB.

## Theoretical background and hypotheses

### Social exchange theory and social identity theory

Social exchange theory holds that an individual’s social behavior is the result of economic and social exchange. Exchange features in reciprocity. When one party does something good for the other, the other party has the obligation or responsibility to do the same in return (Konovsky and Pugh, 1994; Farh et al., 2007; Cropanzano et al., 2017). It reveals that the formation of a continuous, long-term, and complex social relationship between subjects lies in the inter-subject trust, and subjects make decisions under the comprehensive influence of relationship and trust. At present, SET is widely used to study the impact of the relationship between individuals in the workplace and the organizational environment on individual behavior (Farh et al., 2007; Singh and Srivastava, 2016; George et al., 2020). For example, when an organization offers a wide range of educational opportunities to employees, they develop a positive impression about the organization, which, in turn, facilitates employee engagement in social exchange (Kim and Park, 2019).

Social identity theory focuses on answering the questions of “who am I” and “who are we” (Ashforth and Mael, 1989; Van Dick et al., 2006). Social identity is the individual’s realization that he or she belongs to a particular social group, while also recognizing the emotional and value meanings that are brought to him/her as a group member (Van Dick et al., 2006; George et al., 2020; Barattucci et al., 2021). Social identity theory can effectively explain the identity of members of an organization and the resulting attitudes and behaviors (Ashforth and Mael, 1989; Van Dick et al., 2006).

Although social exchange theory and social identity theory provide a theoretical basis for us to correctly explain the employment relationship between individuals and organizations, most of the existing studies regard the two theories as independent ones (Van Knippenberg et al., 2007). Therefore, some scholars have suggested that the two theories should be combined organically during the research process to make some beneficial attempts (Van Knippenberg et al., 2007; Sluss et al., 2008; George et al., 2020). Considering that OCB is influenced not only by external environmental factors, but also by individual values and internal motives, this study combined the two theories in an organic manner, which may better illustrate the bridging role of OI and EL in the process of the effects of OT on OCB.

### Organizational trust

OT is a topic of intense research in the fields of psychology, management, and organizational behavior (Li et al., 2021). Since OT is a complex social and psychological phenomenon, scholars have come to different interpretations of OT from various perspectives (Kmieciak, 2021). Robinson (1996) argues that OT, which is the recognition and trustworthiness of employees in the sincerity and reliability of an organization or leadership, represents employees who identify with the organization and are willing to establish a long-term relationship with the organization. From an interpersonal point of view, Podsakoff et al. (1990) point out that OT is the degree to which employees trust their superiors and colleagues. McAllister (1995) classifies trust into cognitive-based trust and emotional-based trust, depending on the basis on which trust develops. OT is a cognitive judgment about the abilities and reliability of others, and is the result of others’ rational judgment of ability, integrity, impartiality, and other personal qualities based on experience, while emotional trust is the perceptual judgment based on the specific emotions of both parties. OT is an important situational variable in the social exchange relationship (George et al., 2020). When employees perceive that they can benefit from the behavior of the organization or leader, they will positively repay the organization or leader (Yang and Tsai, 2022). Previous studies on OT were mainly carried out in two aspects. (1) The concept, dimensions, and measurement tools of OT were explored from a static perspective (Robinson, 1996). (2) The causes and effects of OT were explored from a dynamic perspective (e.g., Matzler and Renzl, 2006; Berraies et al., 2021; Kmieciak, 2021; Paşamehmetoğlu et al., 2022).

### Organizational trust and employee organizational citizenship behavior

Social exchange theory holds that employees’ attitudes and behaviors depend on the degree of trust and support they receive from the organization (Konovsky and Pugh, 1994; George et al., 2020). Trust is reciprocal (Singh and Srivastava, 2016) and its main function is to promote social exchange relations. The relationship between OT and OCB reflects the social exchange relationship. Trust creates a good working environment for an organization in which employees exhibit more off-role behavior (Yang and Tsai, 2022). Kim and Park (2019), for example, believe that when trust is built between members and the organization, it has a positive impact on the organization and is more likely to show OCB. Studies by Podsakoff et al. (1990) also show that trust is an antecedent variable of OCB and an important factor in employees’ commitment to OCB performance. This study believes that when employees have full trust in the organization and their superiors, they can not only do their work well, but also show OCB. Based on this, the following assumption is also made in this study.

*H1*: Organizational trust positively affects employee’s organizational citizenship behavior.

### Mediating role of organizational identification

OI is an embodiment used by social identity theory in organizational scenarios to reveal employees’ psychological connections with the organization as well as the mechanisms of action (Barattucci et al., 2021). OI plays an important role in modern organization management practice (Marstand et al., 2021). It has positive effects on stabilizing employee turnover tendency, improving employee efficiency, and stimulating innovation consciousness (Ashforth and Mael, 1989). Abrams et al. (1998) point out that organizational identification occurs when employees feel they have a common fate with the organization. Especially in Asian countries that are deeply influenced by Confucian culture, the higher the organizational identity, the higher the expectation of a close connection with the organization (PaRK et al., 2013).

OT is an important factor influencing employees’ identification with the organization. The facilitative effect of OT on organizational identification is supported by previous studies (Kim, 2019). There are studies showing that high levels of leadership cognitive trust can reduce the uncertainty of employees at work, and high levels of leadership emotional trust can provide psycho-social resources that meet the psychological needs of employees, thereby promoting OT (Colquitt et al., 2011). When employees perceive trust in the organization, they are more likely to repay the organization with a positive attitude (such as OI; Kim, 2019; Yang and Tsai, 2022).

OI represents members’ strong sense of identity belonging to the organization and employee perceptions of congruence with the values of the organization (Li et al., 2021). Organizational identification provides an emotional basis for an employee’s OCB, prompting an invisible psychological contract between the employee and the organization. When employees think that they are psychologically connected with the fate of the organization, they will have real dependence and belonging under strong OI, and thus spontaneously make efforts for the development of the organization (Li et al., 2021; Marstand et al., 2021). Studies by Kim (2019) show that OI has a significant effect on OCB. Research by Verburg et al. (2018) also shows that OT helps to improve innovators’ OI, which, in turn, triggers the OCB of innovators. Basing on the above analysis, the following hypothesis has been proposed.

*H2*: OI plays a mediating role between OT and OCB.

### Mediating role of employee loyalty

The frequent turnover of employees brings losses to the enterprise, which not only reduces profits, but also affects the work efficiency and mental state of other employees in the organization. Studies suggest that the cost of recruiting, training, and adapting to a new employee is equivalent to 6–9 months of salary (Beehner and Blackwell, 2016). Therefore, in modern enterprise management, how to retain talents and cultivate employee loyalty has been the focus of researchers and managers. EL is defined as an individual’s identification with the core values philosophy of the organization, which embodies the psychological state of the relationship between the employee and the firm, and is willing to stay in the firm and make a great effort (Meyer and Allen, 1991; Veloso et al., 2021). EL is a concentrated manifestation of psychology and emotion (Dhir et al., 2020).

Previous studies have confirmed that OT is considered an important factor influencing employers’ long-term, effective relationships with employees (George et al., 2020). Employees’ trust in the organization affects their affection and emotions (Matzler and Renzl, 2006; Yang and Tsai, 2022) and is essential to their loyalty and commitment (Altuntas and Baykal, 2010). In organizations lacking in trust, employees tend to have high absence rates, lack of responsibility, and low loyalty. Employees with high loyalty have a higher sense of attachment and belonging, are willing to improve and protect the organization, and have a higher emotional connection. Studies by Matzler and Renzl (2006) show that trust in managers and colleagues can positively affect EL through a mediating effect on employee’s satisfaction. Further, EL can reduce turnover possibility and absenteeism, improve job performance, and promote OCB and voice behavior (Hui et al., 2012; Phuong and Vinh, 2020). The following hypothesis has been proposed.

*H3*: EL acts a mediating role between OT and OCB.

### The chain-mediating role of organizational identification and employee loyalty

According to research literature, we conclude from our review of the previous literature that OT may promote OCB not only through OI (H2) but possibly also by increasing EL (H3). In addition, according to the social identity theory, high OI will narrow the distance between individuals and organizations, make employees’ personal goals consistent with the organizational goals of the enterprise (Ashforth and Mael, 1989), and then affect the EL (Mael and Ashforth, 1992). A follow-up study of 249 samples from 2004 to 2005 by Mignonac et al. (2006) found that perceptions of the organization’s external prestige and need for organizational identification can pose a significantly negative effect on turnover inclination. Studies by Barattucci et al. (2021) also show OI can negatively affect turnover inclination. This study argues that organizational identification emphasizes the process in which individuals integrate themselves with the organization and change from “I” to “we” in self-definition. This type of identification can improve employees’ psychological safety, encourage them to form a sense of dependency on the organization, reduce employee turnover, and enhance their loyalty. The following hypothesis is proposed in this study.

*H4*: In the process of OT affecting OCB of employees, OI and EL play a chain mediating effect.

## Methodology

### Sample and data collection

Through the contact of the research team, we conducted a questionnaire survey and collected relevant data on 8 enterprises (involving finance, education, construction, consulting, and real estate) located in Beijing, Shanghai, Chongqing, and Zhuhai of China. Considering that common method biases may exaggerate the relationship between variables (Yildiz, 2019), we have made some precautions in advance to reduce its impact and to test the causal relationship between variables. First, following the recommendations of Podsakoff et al. (2003), this study used a multistage, multi-source longitudinal study design to conduct a questionnaire at two-time points. Secondly, all the scales employed in the study were the maturity scale, and anonymous was adopted to alleviate raters’ concerns (Elçi and Alpkan, 2009).

The research members actively communicated with the heads of the HR departments of each company, and then they explained the purpose of the questionnaire with each department. Answers on employee basic personal information, OT, and EL were collected in period 1, and 1 month later, the same cohort of employees were invited to continue filling out questionnaires on OI and OCB. A total of 305 valid questionnaires were finally collected by excluding invalid questionnaires, which included scrambled filling in, more than 5 missing data questions, and failed pairing in 2 periods.

Among the surveyed samples, the proportion of men and women were 43.9% and 56.1% respectively, and the proportion of women was higher than that of men. In terms of age, most of the respondents are 21–30 years old and 31–40 years old, 47.2% and 24.9%, respectively, and most of them were front-line employees, which reveals that young people are the backbone of enterprises in this region. From the level of education, respondents with an undergraduate degree or higher were predominant at 60.3%. From the income level, respondents with monthly incomes in the range of 3,001–5,000 RMB as well as 5,001–10,000 RMB were predominant, accounting for ~28.9% and 46.6%, respectively.

### Measures

All constructs were measured using a five-point Likert scale, with response categories ranging from “strongly disagree” (1) to “strongly agree” (5). All constructs were translated into Chinese and the consistency of the translated version was evaluated using the back translation process (Brislin, 1970).

***Organizational trust:*** The McAllister (1995) cognitive trust and emotional trust scales were adopted. Exemplary question is such formulated as “You can trust them to do the main work of a team.” The coefficient of internal consistency of this scale was 0.93 in this investigation.

***Organizational identification:*** In this investigation, the 6-question scale invented by Mael and Ashforth (1992) was adopted. The scale is used in the study of Marstand et al. (2021) and has good reliability and validity. Typical question items include “When someone criticizes my organization, it feels like a personal blame,” and “I’m interested in what other people think of our organization.” The coefficient of internal consistency of this scale was 0.86 in this investigation.

***Employee loyalty:*** In this study, the scale for EL used by Matzler and Renzl (2006) in their study was adopted. It consisted of five questions, such as “I speak positively about my company when I talk to customers” and “I would not immediately switch to another company if I was offered a job.” The coefficient of internal consistency of this scale was 0.92 in this study.

***Organizational citizenship behavior:*** In this investigation, the scale used in the study of Farh et al. (2007) was adopted, which was developed according to Chinese scenarios and was more in line with Chinese local culture. Exemplary question item is such formulated as “Always proactively help new employees adapt to their work environment.” The coefficient of internal consistency of this scale was 0.93 in this investigation.

***Control variables:*** Gender, age, and educational background might affect employee behavior. Following the practice of previous studies, we hired gender, age, and educational background as control variables (Kim, 2019; Yildiz, 2019).

## Data analysis and results

### Test of common method biases

Although a series of measures were taken to ensure the quality of the data during issuing the questionnaires, they may be affected by common method biases because the data were self-rated by respondents. In this way, Harman single-factor test was applied to examine the common method biases (Podsakoff et al., 2003). The results show that after being extracted from exploratory factor analysis results without rotation, there were four factors with characteristic roots larger than 1. The largest variance required factor interpretation was 40.50% (below the 50% threshold), indicating that common method biases had little influence on the study (Podsakoff et al., 2003; Yildiz, 2019).

### Test of the reliability and validity of research variables

Firstly, SPSS24 was employed to analyze the reliability of each variable. The Cronbach’s α coefficient of each measured variable was above 0.86. Assuming that the factor load between measurement questions and potential variables in the model was between 0.65 and 0.85 (above 0.5), Average Variance Extraction (AVE) was between 0.51 and 0.67 (above recommended value 0.5; Paşamehmetoğlu et al., 2022), and combination reliability of potential variables (CR) was between 0.84 and 0.93 (above recommended value 0.7; Fornell and Larcker, 1981), so it indicated that there was a high consistency within variables (see Table 1). Secondly, Amos24 was utilized to test the discriminated validity of the four variables, and the results are shown in Table 2. Four-factor model has the best fitting degree (*χ*^2^ = 553.58, *χ*^2^/df = 1.89, GFI = 0.88, CFI = 0.95, RMSEA = 0.05, TLI = 0.95, IFI = 0.95) and is superior to other alternative models. The original designed model features in excellent discriminated validity.

**Table 1:** Factor loadings of variables and overall reliability.

Variables	Factor loadings	Cronbach’s alpha	Composite reliability (C.R)	Average variance extracted (AVE)
OT	0.79–0.85	0.93	0.93	0.67
OI	0.65–0.74	0.86	0.84	0.51
EL	0.73–0.82	0.92	0.91	0.61
OCB	0.67–0.81	0.93	0.92	0.56

OT, organizational trust; OI, organizational identification; EL, employee loyalty; OCB, organizational citizenship behavior.

**Table 2:** Comparison of measurement model.

Models	Factors	*χ* ^2^	*χ*^2^/df	GFI	CFI	RMSEA	TLI	IFI
Baseline Model	Four Factors: OT, OI, EL, OCB	553.58***	1.89	0.88	0.95	0.05	0.95	0.95
Model 1	Three Factors: OT+ OI, EL, OCB	1037.34***	3.51	0.75	0.86	0.09	0.85	0.86
Model 2	Three Factors: OT, OI, EL + OCB	1270.07***	4.29	0.66	0.82	0.10	0.80	0.82
Model 3	Two Factors: OT + OI, EL + OCB	1753.99***	5.89	0.58	0.73	0.13	0.70	0.73
Model 4	Two Factors: OT + OI + EL, OCB	1918.49***	6.44	0.53	0.70	0.13	0.67	0.70
Model 5	One Factors: OT + OI + EL + OCB	2657.59***	8.89	0.45	0.56	0.16	0.52	0.56

OT, organizational trust; OI, organizational identification; EL, employee loyalty; OCB, organizational citizenship behavior. ***p < 0.001.

### Descriptive statistics

Pearson’s correlation coefficient was adopted to analyze the correlation between variables. Table 3 lists the mean, standard deviation, and correlation coefficients for each variable. As shown in Table 3, OT is significantly positively correlated with OCB (*r* = 0.29, *p* < 0.01). OT is positively correlated with OI (*r* = 0.52, *p* < 0.01). OT is positively correlated with EL (*r* = 0.39, *p* < 0.01). OI is positively correlated with EL (*r* = 0.51, *p* < 0.01), and EL is positively correlated with OCB (*r* = 0.60, *p* < 0.01). OI is positively correlated with OCB (*r* = 0.54, *p* < 0.01). The results preliminarily support the hypothesis proposed in this study and provide the basis for further validation.

**Table 3:** Descriptive statistics and correlations among variables.

Variables	Mean	SD	1	2	3	4	5	6	7
1. Gender	1.56	0.50	1						
2. Age	2.74	0.96	0.09	1					
3. Education	2.57	0.93	0.05	−0.23**	1				
4. OT	3.50	0.83	−0.08	−0.17**	0.01	1			
5. OI	3.65	0.77	−0.06	0.07	0.10	0.52**	1		
6. EL	3.74	0.69	0.09	0.01	0.12*	0.39**	0.51**	1	
7. OCB	3.79	0.74	0.10	0.06	0.15**	0.29**	0.54**	0.60**	1

OT, organizational trust; OI, organizational identification; EL, employee loyalty; OCB, organizational citizenship behavior. **p < 0.01.

### Relationship between organizational trust and organizational citizenship behavior: Test of chain-mediating model

First, we employed the method proposed by Hayes (2013) to test the mediating effect. Model 6 in SPSS Process macro program was utilized for the test and 5,000 repeated samples were set with a confidence level of 95%. Then, OI and EL were examined while keeping gender, age, and educational factors constant, to examine their mediating effects in the process of the effects of OT on OCB. Based on the regression results shown in Table 4, OT has a significant predictive effect on OCB (*β* = 0.32, *t* = 5.92, *p* < 0.001), so H1 is verified. After the mediating variables were introduced, after which, the result indicates that OT plays a significantly positive role for OI (*β* = 0.54, *t* = 11.17, *p* < 0.001); OT has a significant positive effect on EL (*β* = 0.19, *t* = 3.21, *p* < 0.001), OI has a significant positive effect on EL (*β* = 0.41, *t* = 7.03, *p* < 0.001); OI has a positive and significant effect on OCB (*β* = 0.33, *t* = 5.88, *p* < 0.001); EL has a positive and significant effect on OCB (*β* = 0.43, *t* = 8.29, *p* < 0.001). However, the predictive effect of OT on OCB is no longer significant (*β* = −0.03, *t* = −0.62, *p* = 0.54), indicating that OI and EL play a complete mediating role in the impact of OT on OCB.

**Table 4:** Regression analysis.

Outcome variable	Predictor	*R*	*R* ^2^	*F*-value	*β*	*t*-value
OCB		0.37	0.14	12.20		
	Gender				0.10	1.87
	Age				0.15	2.64
	Education				0.18	3.22
	OT				0.32	5.92***
OI		0.56	0.31	33.78		
	Gender				−0.04	−0.85
	Age				0.20	3.98
	Education				0.14	2.82*
	OT				0.54	11.17***
EL		0.55	0.30	25.84		
	Gender				0.13	2.56*
	Age				0.02	0.29
	Education				0.08	1.59
	OT				0.19	3.21***
	OI				0.41	7.03***
OCB		0.67	0.44	39.68		
	Gender				0.07	1.55
	Age				0.04	0.88
	Education				0.07	1.61
	OT				−0.03	−0.62
	OI				0.33	5.88***
	EL				0.43	8.29***

OT, organizational trust; OI, organizational identification; EL, employee loyalty; OCB, organizational citizenship behavior. *p < 0.05; **p < 0.01; ***p < 0.001.

**Figure 1:**
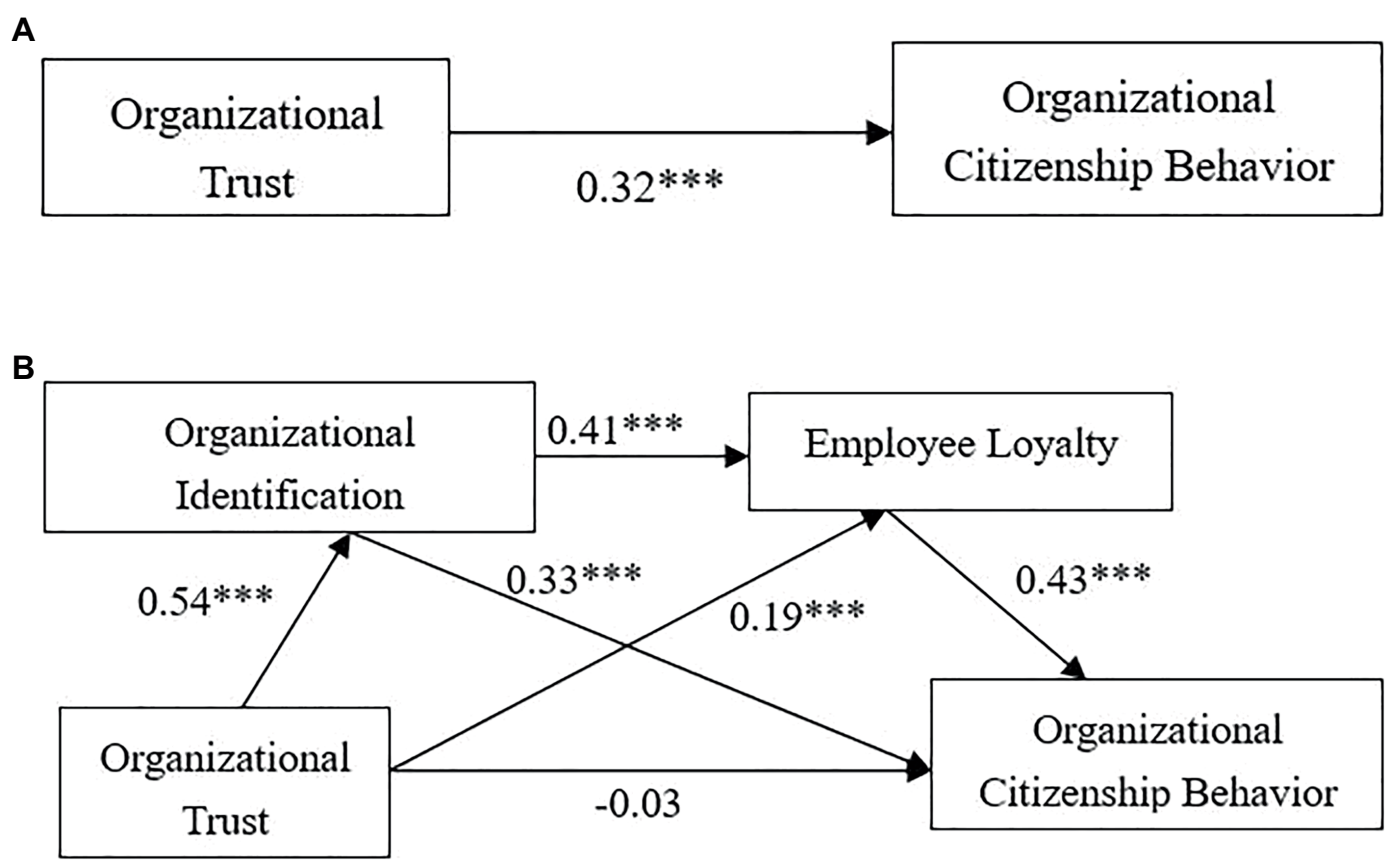
Reflects the direct effect coefficient of OT on OCB **(A)**, and **(B)** represents the path coefficient after adding two mediating variables (OI,EL).

Bootstrap was employed to further test the mediation effect, and the results are shown in Table 5. The mediating effect of OI and EL is significant, and the total mediating effect value is 0.32. Specifically, mediating effect is produced through three multiple mediation processes. First, the indirect effect value of OT → OI → OCB process is 0.16. The upper and lower limits of 95% confidence interval *via* Bootstrap method do not contain 0. OT can predict OCB through OI, so H2 is supported. Second, the indirect effect value of OT → EL → OCB process is 0.07. The upper and lower limits of 95% confidence interval *via* Bootstrap method do not contain 0. OT can predict OCB through EL, so H3 is supported. Finally, the indirect effect value of OT → OI → EL → OCB process is 0.09, and the upper and lower limits of 95% confidence interval *via* Bootstrap method do not contain 0, indicating that the chain-mediating effect is significant, so H4 is supported. The overall empirical results are shown in Figure 1.

**Table 5:** Results of indirect effects.

Paths	Effect	BootSE	95% confidence		
			BootLLCI	BootULCI	%
Total indirect effect	0.32	0.05	0.23	0.41	100.00%
Path1: OT → OI → OCB	0.16	0.03	0.10	0.23	54.88%
Path2: OT → EL → OCB	0.07	0.03	0.02	0.13	32.20%
Path3: OT → OI → EL → OCB	0.09	0.02	0.05	0.13	12.93%

OT, organizational trust; OI, organizational identification; EL, employee loyalty; OCB, organizational citizenship behavior.

## Discussion

How can organizations encourage employees to OCB in the workplace? Which factors promote the identification and loyalty of employees to their organization? This paper claims to make some important conclusions to literature. First, empirical analysis shows that OT can positively promote OCB. Specifically, according to the social exchange theory, a trustworthy organizational environment is conducive to evoking positive emotions of employees, and in return for the organization, employees will do more extra work on their own (Yang and Tsai, 2022). This conclusion supports the previous research conclusions on OT and OCB (Podsakoff et al., 1990; Altuntas and Baykal, 2010; Verburg et al., 2018; Kim and Park, 2019; Yildiz, 2019), as well as the conclusions of Singh and Srivastava (2016) on the relationship between OT and OCB to a certain extent. OCB is a typical extra-role behavior (Singh and Srivastava, 2016). The reason why OT can promote OCB in employees is that a high degree of OT helps to deepen individuals’ understanding and perceptions of organizational scenarios to allow individuals to gain more psychological safety and organizational support at work. Employees’ motivation at workplace is enhanced by perceived organizational trust, which promotes OCB.

Second, organizational identification is important for the work results of the employees (Marstand et al., 2021). Referring to social identity theory, our research concludes that OI mediates between OT and OCB. The more employees perceived trust in the organization, the more employees identify with the organization, which leads to more OCB. This result supports the argument that OT can improve OI in employees raised by previous studies (Colquitt et al., 2011; George et al., 2020), and also supports that OI has a promoting effect on OCB (Van Dick et al., 2006; Kim, 2019). On the basis of the theoretical framework of the Context-Attitude-Behavior Framework, Kim (2019) employed data from a three-time-period questionnaire of 303 Korean employees to find out that OT and OI mediate between job insecurity and OCB. The results of this study are similar.

Finally, EL mediates in the relationship between OT and OCB. Employee’s trust in the organization is one of the major factors affecting EL (Matzler and Renzl, 2006). OT can improve employee satisfaction and engagement, enhance organizational emotional commitment, reduce turnover possibilities, and promote employee to form a good working attitude and behavior (Alshaabani et al., 2022; Yang and Tsai, 2022). It is worth mentioning that this study further supports the view that when employees trust the organization, the higher their loyalty, the more active their work and behavior, and they are more willing to spontaneously conduct OCB. In addition, this study also further confirms the facilitative effect of OI on EL. As OI increases, employees become more loyal.

### Theoretical contributions

The relationship between OT and OCB has been extensively studied over the past 20 years (Yildiz, 2019). Our findings make several contributions to the literature on employees’ OCB. First, it draws on social exchange theory and organizational identity theory to present an integrated model that illustrates how OT directly influences the OI and EL and indirectly influences the employees’ OCB. Based on the context of Chinese companies, the paper further clarifies and deepens the understanding of the relationship between organizational trust and organizational citizenship behavior, and provides fresh insights of social exchange theory and social identity theory in the research field of organizational trust.

Second, we advance the literature on the antecedents of employees’ OCB by explaining organizational identification and employee loyalty as the mediating mechanism that connects OT to employees’ OCB. Previous research suggests that leadership (Barattucci et al., 2021), organizational climate (Marinova et al., 2019), and positive psychological capital (Yildiz, 2019) can affect employees’ OCB. However, little attention has been given to individuals’ identification and loyalty (Sluss et al., 2008). The current study complements the deficiency of existing research on organizational trust and OCB.

Finally, this paper verifies that both organizational identification and employee loyalty can play a mediating role between organizational trust and employees’ OCB. However, employees’ identification is a key factor for employees to engage in OCB, that is, organizational identification can better predict employees’ organizational citizenship behavior than employee loyalty. The results deepened our understanding of organizational identification and employee loyalty on the research of OCB.

### Managerial implications

The current study can provide some understandings for practitioners. Firstly, OT has some important effects on the proper operation and long-term development of organizations, but some organizations do not value building employee trust in the organization. The interaction between members of the organization frontal mainly follows institutional norms (Gould-Williams, 2003). Therefore, enterprises should focus on developing the social exchange relationship between employees and the organization, valuing the interpersonal relationships and trust among employees. For example, managers can demonstrate trust by delegating high-risk tasks to employees to create opportunities for communication with them (Yang and Tsai, 2022). When employees find that their managers trust them, they will put extra effort and ability at work.

Secondly, OI is a psychological bond for maintaining individuals and organizations, which is significant for individuals’ OCB. This study finds that individuals’ identification with an organization is influenced by OT. Therefore, in daily routine, managers should affirm the value of employees and their contribution to the organization, reinforce employee identification, and make employees feel they have an obligation to pay back to the organization. In addition, managers can also establish a good relationship between superiors and subordinates through authorization, guidance, care, and encouragement, which encourages employees to freely express their ideas and timely feedback on their own needs. This will enhance the trust of employees in managers and organizations (Berraies et al., 2021), and enhance the recognition of employees, thus promoting OCB of employees.

Finally, how to retain employees and establish loyalty is a concern for managers worldwide engaged in human resources management (Dutta and Dhir, 2021). Companies usually take various team stabilization measures to improve organizational efficiency and morale. Previously, managers usually increased employee loyalty by offering promotions, increasing salaries, and benefits (Veloso et al., 2021). The results of this study show that OT and OI have significant positive effects on EL. Therefore, in order to attract and retain high-quality, dedicated, and loyal employees, enterprises should create a healthy working environment for them (Dhir et al., 2020), enhance the positive factors to enhance OT, attach importance to fostering a cultural environment with communication, transparency, and inclusiveness, and make employees trust the organization more (Altuntas and Baykal, 2010; Paşamehmetoğlu et al., 2022).

## Limitations and future direction

This study, although obtaining some important conclusions on the study of the OCB effects of OT on employees, still has some limitations and deficiencies, which are mainly reflected in the following aspects: (1) Employee behavior is different in different cultural backgrounds. Since the study sample comes from Chinese enterprises, the applicability of the results is limited. Therefore, future research can be supplemented from a multicultural perspective. (2) The data of this study were all self-rated by the respondents. Although the common biases of the data were examined after the collection and the results were within reasonable limits, the errors caused by the common biases cannot be completely ruled out. To guarantee that the data are more scientific and rigorous, in future studies, the evaluation of managers could be introduced in the source of the data to optimize the study design. (3) The study of OCB is complex. This paper only explored the effects of OT on OCB, and its antecedent variables and influencing mechanisms remain to be uncovered and enriched. For example, future research could further discuss whether organizational climate and organizational culture could indirectly affect OCB through OI and EL.

## Conclusion

Employing a two-wave and multi-source strategy to conduct hypothesis validation with 305 validated questionnaires from Chinese enterprises, this empirical research finds: (1) OT has a significant effect on OCB; (2) OI plays a mediating role between OT and OCB; (3) EL plays a mediating role between OT and OCB; and (4) OI and EL have a significant chain-mediating role between OT and OCB.

## Data availability statement

The raw data supporting the conclusions of this article will be made available by the authors, without undue reservation.

## Ethics statement

Ethical review and approval was not required for the study of human participants in accordance with the local legislation and institutional requirements. Written informed consent from the patients/participants was not required to participate in this study in accordance with the national legislation and the institutional requirements.

## Author contributions

All authors contributed to the study conception and design. Material preparation, data collection, and analysis were performed by YD, YT, WC, and JH. The first draft of the manuscript was written by YD. All authors contributed to the article and approved the submitted version.

## Funding

This work was supported by the National Social Science Foundation (21FYYB056), and JH (houjie918@163.com), School of Foreign Languages, Huaibei Normal University (235000).

## Conflict of interest

The authors declare that the research was conducted in the absence of any commercial or financial relationships that could be construed as a potential conflict of interest.

The reviewer CW and GH declared a past co-authorship with the author YT to the handling editor.

## Publisher’s note

All claims expressed in this article are solely those of the authors and do not necessarily represent those of their affiliated organizations, or those of the publisher, the editors and the reviewers. Any product that may be evaluated in this article, or claim that may be made by its manufacturer, is not guaranteed or endorsed by the publisher.
